# Ultra-orphan diseases: A cross-sectional quantitative analysis of the natural history of isolated sulfite oxidase deficiency

**DOI:** 10.1371/journal.pone.0323043

**Published:** 2025-05-29

**Authors:** Laura Göde, Matthias Zielonka, Sven F. Garbade, Roland Posset

**Affiliations:** Medical Faculty Heidelberg, and Center for Pediatric and Adolescent Medicine, Department I, Division of Pediatric Neurology and Metabolic Medicine, University Hospital Heidelberg, Heidelberg University, Im Neuenheimer Feld 430, Heidelberg, Germany; University of Delhi, INDIA

## Abstract

**Objective:**

Isolated sulfite oxidase deficiency (ISOD; OMIM #272300) is a devastating rare neurometabolic disorder due to biallelic pathogenic variants in the *SUOX* gene, that typically results in neonatal refractory epilepsy and progressive severe encephalopathy. Knowledge on the quantitative natural history of ISOD is limited and clinical outcome parameters for future clinical trials remain to be defined.

**Material and methods:**

We performed a comprehensive analysis of published cases (N=74) with ISOD applying quantitative retrospective natural history modeling (QUARNAM). Main outcome parameters were age of disease onset, diagnostic delay and survival. Clinical characteristics and potential associations between biochemical parameters and clinical outcome (i.e. age of disease onset, survival) were explored.

**Results:**

The median survival period of the study cohort was 60 months. ISOD typically presented shortly after birth with a median age of onset of 3 days. Median age at diagnosis was 10 months, leading to a substantial median diagnostic delay of 5.7 months. Homocysteine concentrations in plasma correlated with age of disease onset. An association of biochemical parameters of cysteine metabolism and survival could not be identified.

**Conclusion:**

The present analysis describes long-term outcome measures adding to the quantitative understanding of the natural history of ISOD, which might be helpful in the planning of prospective clinical trials and potentially stimulate development of targeted therapies in the future.

## Introduction

Isolated sulfite oxidase deficiency (ISOD; OMIM #272300) is an ultra-rare autosomal recessive neurometabolic disorder caused by biallelic pathogenic variants in the *SUOX* gene located on chromosome 12q13.2 [1,2]. The enzyme sulfite oxidase is involved in the detoxification of highly reactive sulfite to sulfate, which mainly is derived from catabolism of sulfur amino acids such as methionine and cysteine. Deficiency of sulfite oxidase leads to an excessive accumulation of sulfite, causing bioenergetic dysfunction and severe brain damage [3–6]. Moreover, sulfite-induced cleavage of disulfide bonds to form S-sulfocysteine from cysteine exerts excitotoxic effects via NMDA-receptors [7,8]. Clinical presentation of ISOD can vary in terms of onset and severity ranging from a life-threatening early-onset within the first days of life with therapy-refractory seizures, feeding difficulties and rapidly progressive encephalopathy to an attenuated late-onset disease form with developmental delay or regression as well as movement disorders with intermittent or progressive symptoms [9–12]. Lens dislocation or subluxation develops in a significant proportion of individuals surviving the newborn period [9]. Systemic manifestations are rare and may include pyloric stenosis [9].

The precise prevalence and incidence of ISOD are unknown. Orphanet currently lists 50 known cases [13]. To date, there are no clinical trials in humans listed on clinicaltrials.gov (accessed 15 May 2024; Search for: isolated sulfite oxidase deficiency | Card Results | ClinicalTrials.gov). Moreover, despite ongoing research efforts in recent years, there was no orphan drug designation granted for any compound intended to treat ISOD by the Food and Drug Administration thus far [14]. Quantitative characteristics especially with regard to relevant (“hard”) clinical outcome parameters such as estimated survival rates of individuals with ISOD are limited, thereby hampering our understanding of the natural disease course of ISOD. However, reliable quantitative data of the survival of untreated individuals is indispensable to assess the long-term effect of potential novel therapeutic approaches as benchmark for future interventional trials. Diagnostic suspicion of ISOD is rendered difficult by the rarity of this metabolic disorder and establishing the diagnosis requires specialized assays that are not available in routine care setting, which might bear the risk of considerable diagnostic delay. However, as soon as novel therapies such as gene replacement therapies are developed, raising disease awareness to shorten the diagnostic process would be indispensable in light of the concept of a limited window of opportunity for clinical improvement after early intervention. Conducting an international prospective natural history study would be virtually infeasible given the rarity of the disease. Important quantitative data especially on relevant clinical endpoints in ultra-orphan neurogenetic diseases can be timely and efficiently obtained from systematic analyses of published case reports and case series [15–20]. We therefore directed our efforts to quantitatively analyze the natural history of ISOD, i.e., disease onset, diagnostic delay and survival based on the yet-to-be-explored published evidence by applying quantitative natural history modeling (QUARNAM) [21]. Moreover, clinical characteristics and potential associations of biochemical parameters of sulfite metabolism and clinical outcome as well as the geographical distribution of probands were assessed, which may inform future clinical trials.

## Materials and methods

The present analysis was performed in accordance with STROBE (STrengthening the Reporting of OBservational studies in Epidemiology) criteria (http://www.strobe-statement.org) [22]. The research strategy of QUARNAM was previously described in detail [21]. Briefly, based on a comprehensive literature research on PubMed clinical descriptions or case series were identified following extraction of relevant clinical, biochemical and/or genetic data on an individual proband level. Data was systematically assessed to further delineate major quantitative disease characteristics such as age of disease onset and age at diagnosis, diagnostic delay, estimated survival, as well as potential correlations between biochemical parameters and clinical outcome (i.e., age of disease onset, survival). Moreover, relative frequency of disease features, EEG abnormalities and morphological alterations in cranial imaging were investigated for further phenotypic characterization. Information on global distribution of individuals with ISOD was collected. A more detailed description on literature review, definition of variables and statistical analyses is provided below.

### Literature review and definition of variables

We conducted a comprehensive literature search on PubMed using the keywords “isolated sulfite oxidase deficiency”, “isolated sulphite oxidase deficiency”, “ISOD”, “SUOX deficiency” and “sulfocysteinuria” as search terms. Identified publications were downloaded and manually sorted for reports containing relevant clinical, biochemical and/or genetic information. Reports qualifying for data extraction were published between 1967 and 2022. In total, we identified 74 individuals out of 71 publications with sufficient information for further quantitative analysis (S1 Fig). The articles were published in English, French and Spanish. Close of database was 31 October 2023. The inventory of publications on individuals with ISOD is illustrated in S1 Table.

The following variables were extracted: age of onset, age at diagnosis, symptoms leading to diagnosis, mode of diagnosis, last reported age, parental consanguinity, reported variants in the *SUOX* gene, information on whether the proband is alive or deceased, S-sulfocysteine in plasma, cystine in plasma, cysteine in plasma, taurine in plasma, total homocysteine in plasma, S-sulfocysteine in urine, sulfite in urine, thiosulfate in urine, taurine in urine, sulfite oxidase activity in fibroblasts or hepatocytes, publication year and origin of patients. Of note, only unequivocal genetic test results were included in the analysis. In analogy to previous studies, age of onset refers to the timepoint of occurring first symptoms attributed to ISOD, whereas age at diagnosis was determined as timepoint of first biochemical, enzymatic or genetic evidence/confirmation of ISOD as underlying diagnosis as reported in the respective case studies. Individuals were considered to be alive at the time of publication if it was not explicitly stated that the proband was deceased. If the origin of the proband has not been explicitly stated in the report, the country of proband’s origin has been attributed to the country of the first author’s institutional affiliation in the respective case description. If information regarding time was stated in semiquantitative terms in the respective publications, we took a conservative approach and defined the findings as follows: “at birth” or “soon after birth” = day 1, “postnatal period” = day 28, “fourth week of life“ = day 28, “first month of life” = day 30, postmortem” = age at death.

### Statistical analysis

Techniques of descriptive statistics were applied as previously reported [15,19,20]. Baseline demographics were summarized using counts and percentages of the total study cohort. Since biochemical parameters in varying specimens were measured in various laboratories, values were expressed as percentage of the mean of the reference interval. Correlation between two numeric variables were computed with Pearson product-moment correlation. Survival was estimated with the Kaplan-Meier method. Patient data were censored at the time of last follow-up if the patient was still alive at last description based on data gathered from each publication. The log-rank test was applied to compare potential differences between subgroups. Diagnostic delay was calculated as difference between age at diagnosis and age of disease onset. To evaluate the relationship between biochemical parameters with age at disease onset or survival, linear regression and Cox proportional hazard models were applied. Unbiased recursive partitioning was applied to identify potential threshold values for the differentiation of subgroups in potential associations of biochemical parameters with clinical outcome measures [23]. To assess whether there exist distinct disease subtypes in ISOD or whether there is rather a continuous spectrum of clinical phenotypes, a cluster analysis of reported signs and symptoms (yes/no coded) was performed applying the following work algorithm: 1) For each included individual, it was coded if a sign or symptom was present or not. 2) Binary distance measure (‘Jacquard’ distance) was computed to reveal a dissimilarity matrix. 3) Hierarchical cluster analysis with Ward-like fusion algorithm was applied to the previously computed dissimilarity matrix to group individuals. 4) Data was illustrated as dendrogram depicting the agglomeration error when clustering individuals into groups. 5) Two groups of individuals were identified with a comparable set of signs and symptoms in varying frequency of occurrence. R function ‘dist(method=”binary”)’ was used to compute the dissimilarity matrix, function ‘hclust(method=”Ward.D”)’ to compute clusters and ‘cutree(k=2)’ to extract two groups representing disease subtypes. Results of the cluster analysis were visualized with a dendrogram [24]. The presence in percentages of each sign or symptom per group was visualized by a line chart. The world map was plotted using the R extension “ggmap” [25]. Missing data were not imputed. Sensitivity analyses were not conducted. All analyses were performed using R (http://www.r-project.org) version 4.4.0. P values reported were two-sided. P ≤ 0.05 was considered statistically significant.

### Ethics declaration

As the present analysis does not contain any identifying personal information, a formal ethics approval was not required.

## Results

Overall, we identified 74 patients out of 69 families (published between 1967 and 2022) for further statistical analysis. Baseline demographics of the study cohort are depicted in Table 1. The origin of afflicted individuals is illustrated in S2 Fig. Probands had to be excluded from specific subanalyses due to missing data in the corresponding subsets. Sample sizes (N) are indicated for each analysis.

**Table 1:** Characteristics of the study cohort of individuals with ISOD.

Population characteristics	N	Percentage
*Sex* (*N* = 74)		
Female	33	44.6
Male	38	51.4
Not reported	3	4.0
*Mode of diagnosis *(*N = 74*)		
Biochemical	8	10.8
Biochemical & enzymatic	12	16.2
Biochemical & enzymatic & genetic	10	13.5
Biochemical & genetic	39	52.7
Genetic	5	6.8
*Alive at last follow-up *(*N = 74*)		
Yes	45	60.8
No	29	39.2
*Families with* (*N = 69*)		
One affected offspring	66	95.7
Two affected offsprings	1	1.4
Three affected offsprings	2	2.9
*Parental consanguinity (N = 66)* Yes No	25 41	37.9 62.1

**Fig 1:**
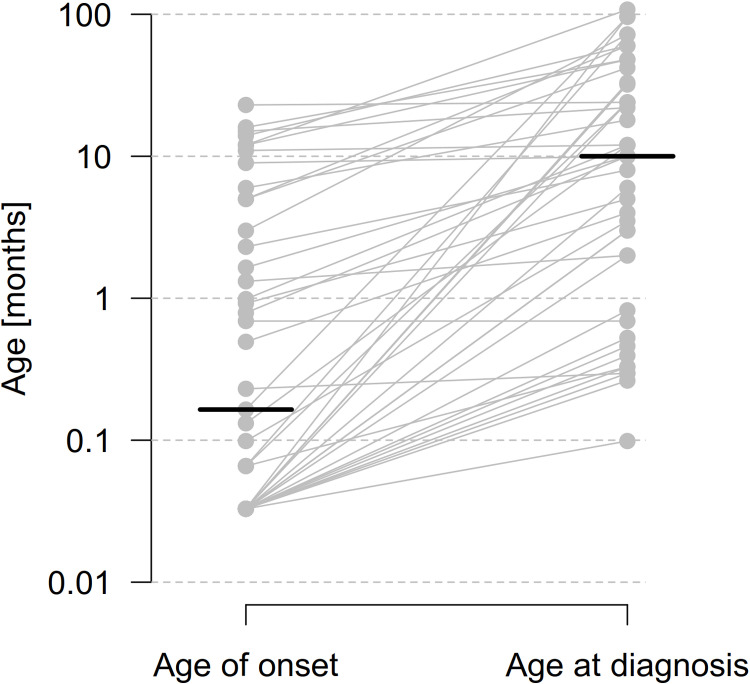
Age of disease onset and age at diagnosis for individuals with ISOD. Median age of onset was 3 days (N = 72). Median age at diagnosis was 10 months (N = 44), leading to a substantial diagnostic delay of 5.7 months in median (N = 43). Horizontal lines indicate the medians for age of onset and age at diagnosis. The slopes of connecting lines represent the diagnostic delay between onset of the disease and time of diagnosis. Please note the logarithmic scale of the ordinate. ISOD; isolated sulfite oxidase deficiency.

In the first step, we quantitatively analyzed age of disease onset and age at diagnosis for the overall study cohort to determine diagnostic delay as surrogate parameter for disease awareness in this ultra-rare neurogenetic condition. Median age of disease onset was 3 days, interquartile range (IQR) from 1 to 28 days. In contrast, median age at diagnosis was 10 months, IQR from 1.7 to 35.3 months, thereby leading to a median diagnostic delay of 5.7 months with an IQR from 0.7 to 32 months (**Fig 1**).

**Fig 2:**
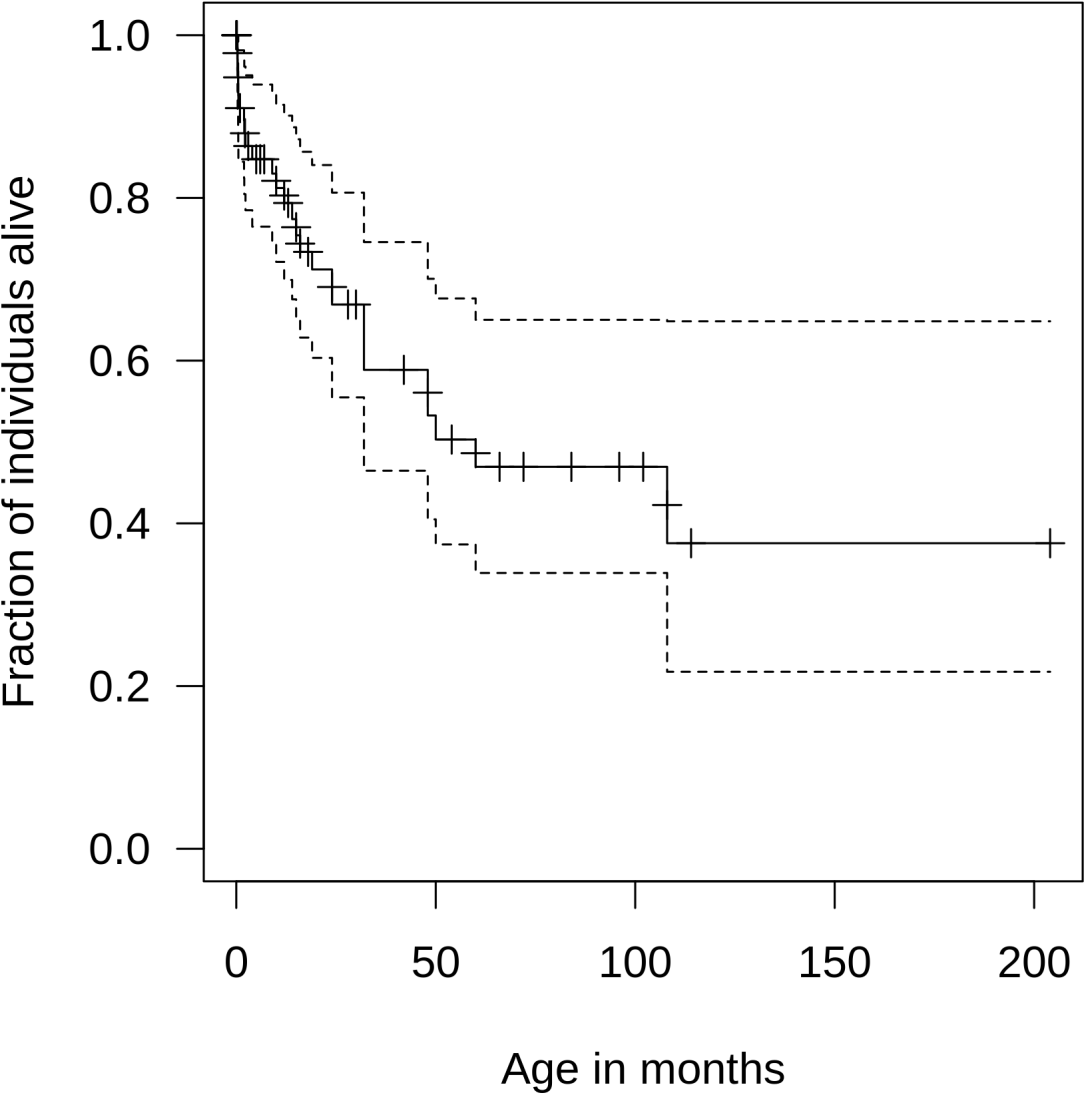
Kaplan-Meier estimated survival rates for individuals with ISOD. Median survival of individuals within the study sample was 60 months (N = 72). Black line indicates the estimated survival curve. Dashed lines illustrate the 95% confidence interval of estimated survival. Censored individuals, who were alive and lost to follow-up, are marked with a “+” at last reported age. ISOD; isolated sulfite oxidase deficiency.

**Fig 3:**
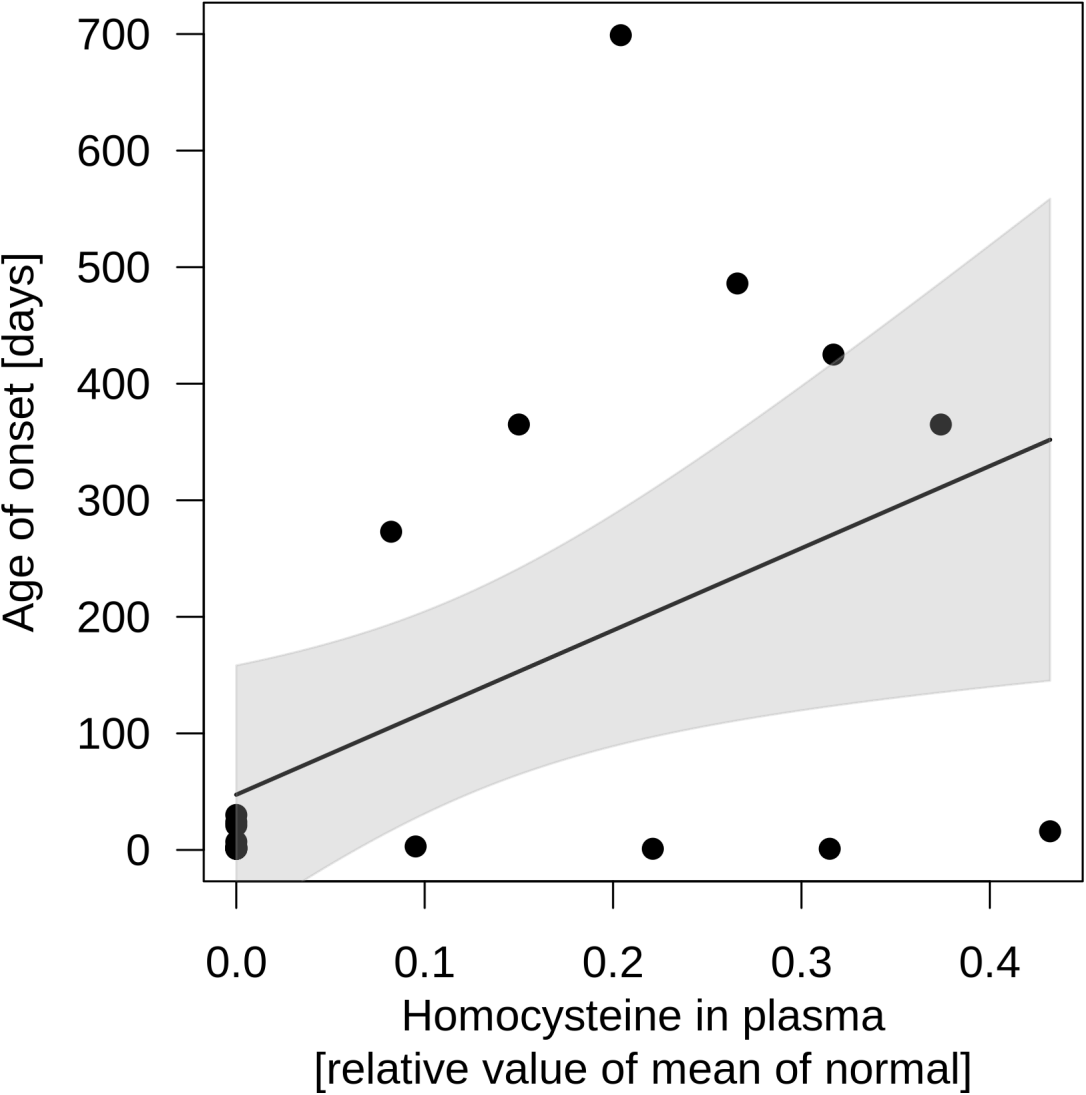
Age of disease onset in individuals with ISOD subject to homocysteine concentration in plasma. Total homocysteine concentration is positively associated with the age of disease onset (Linear regression: r_PM_ = 0,49; Intercept = 47,39, p = 0,382; slope β_Homocysteine plasma_ = 704,96, p = 0.023). Each point represents a single proband (N = 21). Age of onset is illustrated in days. Homocysteine concentration is represented as relative value of the mean of the respective reference range reported in the individual case description. Black line displays the estimated regression curve, gray shading indicates the confidence interval.

We next investigated survival rates by applying the Kaplan-Meier method. Median estimated survival of individuals with ISOD was 60 months (lower limit of 95% confidence interval (CI) 32 months, upper limit of CI could not be determined; Fig 2). At last report, approximately 60% of affected children and adolescents were alive, while approximately 40% deceased during the disease course within 200 months (Table 1). Cox proportional hazard analyses of investigated biochemical parameters such as S-sulfocysteine in plasma, cystine in plasma, cysteine in plasma, taurine in plasma, S-sulfocysteine in urine, sulfite in urine, thiosulfate in urine, and taurine in urine could not identify an association with survival for the prediction of survival rates in the study cohort. Intriguingly, while total homocysteine concentration in plasma was not associated with survival, a positive association with age of disease onset could be identified (Correlation r_PM_ = 0,49; slope β_Homocysteine plasma_ = 704.96; p = 0.023; linear regression; Fig 3). In contrast, an effect of sulfite oxidase activity in fibroblasts or hepatocytes on survival or age of disease onset could not be detected. Moreover, a correlation between sulfite oxidase activity (in fibroblasts or hepatocytes) as well as total homocysteine concentration in plasma and biomarkers of cysteine metabolism (S-sulfocysteine, thiosulfate) was not observed. Dispersion measures of biochemical parameters are graphically represented in S3 Fig. Descriptive characteristics of biochemical variables and last reported age are depicted in S2 Table.

In the present cohort, 36 unequivocal pathogenic variants in the *SUOX* gene were reported in 39 individuals with the majority (N = 25/39) occurring in a homozygous state as private familial disease variants (S3 Table). A clear-cut genotype-phenotype correlation was not apparent.

**Fig 4:**
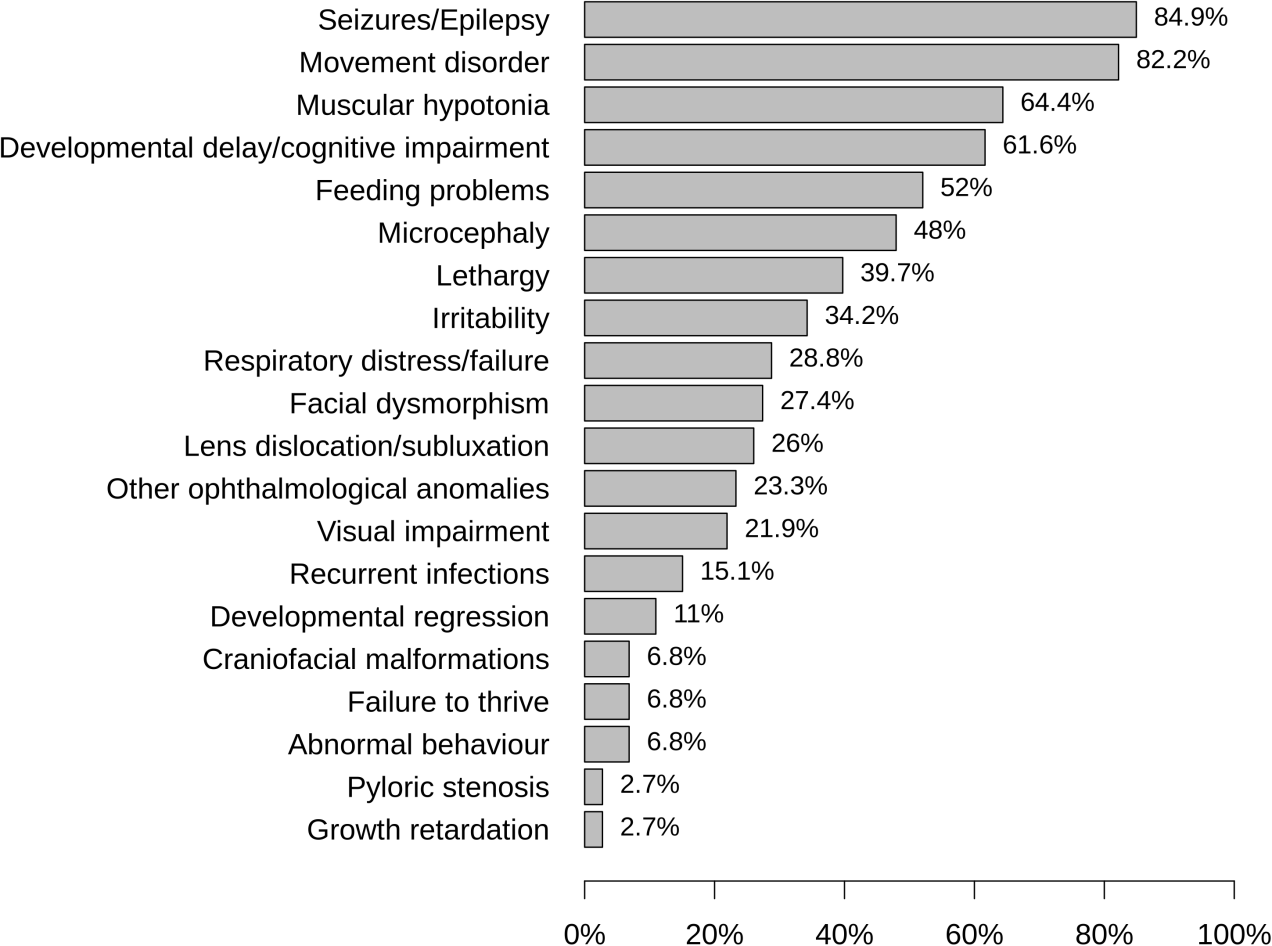
Relative frequency of presenting signs or symptoms in the study cohort of individuals with ISOD. In individual probands two or more signs or symptoms may have been reported (N = 74). ISOD; isolated sulfite oxidase deficiency.

We analyzed the distribution and frequency of presenting or leading signs and symptoms in the study cohort (**Fig 4**). The most common features of ISOD in descending order of frequency were epilepsy/seizures, movement disorders, muscular hypotonia, developmental delay/cognitive impairment, feeding problems, microcephaly, reduced consciousness/lethargy, irritability, respiratory failure, facial dysmorphism and lens dislocation/subluxation. Intriguingly, lens dislocation/subluxation was reported to occur bilaterally in the majority of cases with a median age of diagnosis of 15 months (IQR from 8.5 to 39.6 months). In three individuals (4.1% of the overall study cohort) prenatal anomalies were reported. Prenatal symptoms included weak fetal movements (N = 2/3), polyhydramnios (N = 1/3) and possible fetal seizures (N = 1/3). Prenatal cranial ultrasound and/or MRI were performed in three occasions and showed mega cisterna magna (N = 2/3), corpus callosum thinning (N = 1/3), enlarged ventricles (N = 1/3), a cystic structure (N = 1/3), as well as white matter and basal ganglia abnormalities (N = 1/3). These radiological findings are consistent with postnatal radiological findings, indicating that ISOD might have a prenatal disease onset. In seven families with at least one affected child, prenatal diagnosis was performed in subsequent pregnancies. In two occasions a prenatal diagnosis of ISOD was made and both pregnancies ended in termination.

Clinical feature clustering is depicted in S4 Fig. Ward fusion algorithm allowed the mathematical identification of two statistically different subgroups of clinical presentation in ISOD, which is in line with previous observations [1,2]. Those subgroups exhibit similar disease features in varying frequency of occurrence in each group. The results of comparable clinical pattern in both groups imply a phenotypic spectrum of increasing phenotypic severity in ISOD (S4 Fig). Cranial magnetic resonance imaging (MRI) was reported in 53 individuals. The most common pathologies consist in descending order of frequency of white matter abnormalities, basal ganglia/thalamus anomalies, brain atrophy, cystic lesions, widening of cerebrospinal fluid spaces, corpus callosum hypoplasia/thinning, hemorrhagic changes, cerebral edema, calcifications and fossa posterior anomalies (S5 Fig). EEG anomalies were reported in 86.5% of individuals with ISOD (N = 32/37).

## Discussion

We quantitatively defined the natural history of the ultra-rare neurometabolic disorder ISOD in a global cohort of 74 individuals, in particular relevant clinical outcome parameters including age of disease onset, estimated survival and seminal clinical features, and delineated a correlation of homocysteine concentration in plasma with age of disease onset. Given that Orphanet has listed 50 known cases of ISOD as by October 2024 [13], the present analysis represents the largest cohort studied thus far.

As in other orphan neurogenetic conditions [16–20], the diagnostic delay in ISOD is substantial with a median diagnostic delay of 5.7 months after disease onset at day 3 in median. Obstacles for a timely diagnosis may be (1) low disease awareness due to the rarity of the disease, which might be primarily considered as differential diagnosis by clinicians with expertise in the field of inherited metabolic disorders, (2) access to specialized facilities for metabolic and/or genetic investigations, and (3) due to the fact that there are currently no causal treatment options available to address the underlying pathomechanisms associated with disease manifestation and progression and therefore ISOD might not be prioritized in the diagnostic process. However, early diagnosis is important for counseling of affected families in order to minimize uncertainty and help to make informed decisions in clinical care and family planning. Moreover, as soon as causal therapies (e.g., RNA or gene delivery) are available, early diagnosis might be crucial for timely treatment initiation that might potentially be associated with prevention, disease stability, (partial) reversibility, or improved survival, as shown in other ultra-rare conditions [26].

Pattern recognition is important for diagnostic suspicion of ISOD. The differential diagnosis should be considered once there is a clinical pattern of therapy-refractory seizures, movement disorders, muscular hypotonia, developmental delay/cognitive impairment, feeding difficulties, microcephaly, lethargy or irritability. Notably, lens dislocation/subluxation was reported in 26% of cases with a median age at diagnosis of 15 months. It is therefore suggested to include ISOD as differential diagnosis in etiologic workup of (early-onset) lens dislocation, especially in the presence of additional neurologic symptoms [9,27]. Characteristic MRI features were white matter abnormalities, basal ganglia/thalamus anomalies, brain atrophy and cystic lesions. The clinical and morphological disease characteristics of ISOD in the present study corroborate and expand the data published by Tan and coworkers [12] as well as Claerhout and colleagues [10].

A classification into two specific subtypes was proposed for ISOD [1,2,28]. This observation is corroborated by the present study. However, both groups exhibit similar neurological disease features with a varying degree of frequency, which implies a continuous phenotypic spectrum of increasing phenotypic severity in ISOD. This observation is in accordance with other rare neurogenetic disorders such as Farber disease, free sialic acid storage disease, Gaucher disease or Wolfram syndrome 1 [18,19,29,30].

In case of clinical suspicion of ISOD, especially in case of intractable neonatal seizures diagnostic workup should start with a urine sulfite-dip stick, that is widely available in clinical routine. A positive urine dip-stick should further prompt biochemical investigations as it cannot confirm or rule out the diagnosis of a sulfite intoxication disorder. Quantification of S-sulfocysteine in urine is currently the most reliable and valid laboratory marker of sulfite accumulation and should be promptly used to biochemically confirm a suspected inherited sulfite intoxication disorder [2]. Since increased sulfite concentrations cause a decrease of homocysteine levels [31,32], quantification of total homocysteine in plasma is another alternative diagnostic approach to provide reliable indirect evidence of sulfite accumulation in case of reduced plasma concentrations of homocysteine [2].

Molybdenum cofactor deficiency (MoCD) is an important differential diagnosis of ISOD, as the phenotypical presentations are largely overlapping and cannot be reliably distinguished by clinical examination [2,16,33]. Since substitution with synthetic cyclic pyranopterine monophosphate (cPMP, fosdenopterin) has emerged as novel therapeutic strategy for MoCD type A and was granted market authorization by the FDA in 2021 and by the European Medicines Agency in 2022, early identification of individuals with MoCD is considered crucial for improved outcomes in MoCD type A [2]. In contrast to ISOD, MoCD is associated with specific purine abnormalities serving as primary biomarkers for MoCD [34]. Absence of molybdenum cofactor inactivates xanthine oxidase resulting in a remarkable accumulation of hypoxanthine and xanthine, whereas uric acid in urine or plasma is typically decreased or undetectable [33]. Therefore, quantification of purines in urine is an indispensable investigation to biochemically differentiate between ISOD and MoCD [2]. Genetic confirmation of ISOD is achieved by Sanger-sequencing of the *SUOX* gene.

Currently, there are no clinical trials for ISOD in humans listed on clinicaltrials.gov (accessed 15 May 2024). No orphan drug designation was granted by the FDA for any drug intended to treat ISOD [14]. Dietary sulfur restriction with low methionine and cysteine intake has been proposed for the treatment of ISOD and was reported to provide clinical benefit for some individuals with an attenuated disease course. A convincing clinical benefit for individuals with a severe phenotypic presentation has not been consistently found [2]. Intriguingly, bezafibrate, an agonist of peroxisome proliferator-activated receptors, was shown to mitigate and prevent mitochondrial dysfunction, glial reactivity and neuronal damage in a rat model of cerebral sulfite accumulation, which might have future implications for the treatment of ISOD [35,36]. The survival data in the natural history can serve as a valuable benchmark for the assessment of long-term efficacy of potential targeted therapies in the future. Intriguingly, total homocysteine concentration in plasma was associated with the age of disease onset. Individuals with ISOD and (very) low homocysteine levels showed first symptoms of ISOD earlier than individuals with higher homocysteine values. However, in contrast to other neurogenetic diseases [17–20], an association of biochemical parameters with regard to survival could not be detected, potentially due to low data density for specific biochemical parameters in the present sample. It is tempting to speculate, that increased sample sizes of biochemical parameters of sulfite metabolism might identify further associations with clinical outcomes in ISOD. Assuming sulfite oxidase deficiency as common underlying pathomechanistic factor for the clinical presentation in both ISOD and MoCD, it can be hypothesized that biomarker-outcome correlations identified in ISOD might potentially also be detected in MoCD. Two large retrospective studies provided important insights into genetic, biochemical and clinical characteristics of individuals with MoCD including S-sulfocysteine concentrations. However, potential associations of biochemical parameters (e.g., homocysteine, S-sulfocysteine, thiosulfate) with clinical endpoints such as age of onset or survival were not investigated [16,34].

Global distribution of individuals with ISOD - as depicted in the present analysis - was panethnic and might be helpful for the planning and conductance of future prospective clinical trials. Importantly, innovative trial designs – such as but not limited to basket clinical trials – are currently being developed (e.g., by the Innovative Health Initiative) especially with a focus on rare disease research that establish reusable framework and tools to facilitate the setup and implementation of patient-centered collaborative platform trials in Europe ultimately increasing trial feasibility and access for affected individuals, that might be of importance for future drug development in ISOD [37].

## Limitations and directions for future research

QUARNAM was previously applied in other rare neurogenetic conditions, when prospective natural history studies are virtually not feasible or difficult to perform [15–20]. This methodological approach has some inherent limitations: First, survival data are at least in part historical and survival may change over time due to improved clinical care. Given that there is currently no causal treatment available to (effectively) address the underlying pathomechanisms associated with disease manifestation and progression in ISOD, potential bias for survival data might be introduced by improved supportive clinical care over time (e.g., antiepileptic medication, intensive care protocols), that cannot be corrected for in the present analysis. Moreover, improved supportive care as confounding factor that potentially hampers the establishment of clear correlations between biomarkers and clinical outcomes cannot be excluded. Second, ascertainment bias and missing data due to the lack of a standardized study protocol results in difficulties to assess softer clinical endpoints such as clinical features. Third, laboratory data were pooled and analyzed according to established standards [15–20]. Data reported in this analysis should be regarded exploratory and might need to be confirmed in future protocol-driven natural history studies (including disease registries), that require the availability of resources in terms of time and personnel. Moreover, it remains to be elucidated in future research whether associations of biochemical parameters with clinical outcome identified in ISOD might also apply for MoCD assuming the shared molecular pathomechanism of sulfite oxidase deficiency with subsequent alterations in sulfur metabolism. Given the above limitations, QUARNAM was the most rapid and feasible method to describe important quantitative outcome measures that will be helpful in the planning of future clinical trials in this ultra-rare condition. Potentially, improved quantitative understanding of the natural history of ISOD might stimulate and accelerate the development of specific therapies [38].

## Conclusion

The present cross-sectional, retrospective analysis describes the quantitative natural history including relevant clinical outcome parameters in a global cohort of 74 individuals with ISOD. The results might inform future prospective clinical trials and potentially stimulate the development of novel targeted therapies.

## Supporting information

S1 Fig Study flow diagram: Identification of clinical case reports with ISOD. The reports analyzed were published up to 31 October 2023. ISOD, isolated sulfite oxidase deficiency.
(JPG)

S2 Fig Geographical distribution pattern of individuals with ISOD. ISOD exhibited a panethnic distribution with hotspots in China, Saudi Arabia, Canada and the USA. Blue scales indicate the number of affected individuals per country. ISOD, isolated sulfite oxidase deficiency.
(TIF)

S3 Fig Descriptive characteristics of biomarkers of cysteine metabolism in individuals with ISOD. Numeric values of the ordinate refer to fold-change of mean of the respective reference range as relative values for each parameter. (A) Biochemical parameters as determined in plasma. (B) Biochemial parameters as determined in urine. For biochemical parameters the following outliers were not visualized due to reasons of graphical clarity: S-sulfocytsteine: 170; sulfite: 31; thiosulfate: 260; taurine: 20. Data are shown as median (black thick line) and mean (triangle), length of the box corresponds to the interquartile range (IQR), upper and lower whiskers correspond to max. 1.5 x IQR. AASA, α-aminoadipic semialdehyde; IQR, interquartile range; ISOD, isolated sulfite oxidase deficiency.
(TIF)

S4 Fig Clinical feature clustering of individuals with ISOD. (A) Dendrogram: Clinical symptom clustering. The dendrogram enabled the grouping of clinical features into two groups. (B) Relativ frequency of symptoms per group. The two mathematically defined groups exhibited similar disease features in varying frequency, indicating a spectrum of increasing phenotypic severity in ISOD. ISOD, isolated sulfite oxidase deficiency.
(TIF)

S5 Fig Relative frequency of reported pathologies in cranial magnetic resonance imaging in individuals with ISOD. In individual probands two or more signs or symptoms may have been reported (N = 53). ISOD; isolated sulfite oxidase deficiency.
(TIF)

S1 Table Inventory of publications on individuals with ISOD included in the present analysis. (DOCX)

S2 Table Biochemical and survival characteristics of the ISOD study population. (DOCX)

S3 Table Reported genotypes of individuals with ISOD (N = 39). (DOCX)

## Abbreviations:

IQR: interquartile range
ISOD: isolated sulfite oxidase deficiency
MoCD: molybdenum cofactor deficiency
QUARNAM: quantitative natural history modeling
