# Supplementary figures and images for: Ultra-orphan diseases: A cross-sectional quantitative analysis of the natural history of isolated sulfite oxidase deficiency
Source: PLoS One. 2025 May 29;20(5):e0323043. doi: 10.1371/journal.pone.0323043 (PMC12122042; doi:10.1371/journal.pone.0323043)

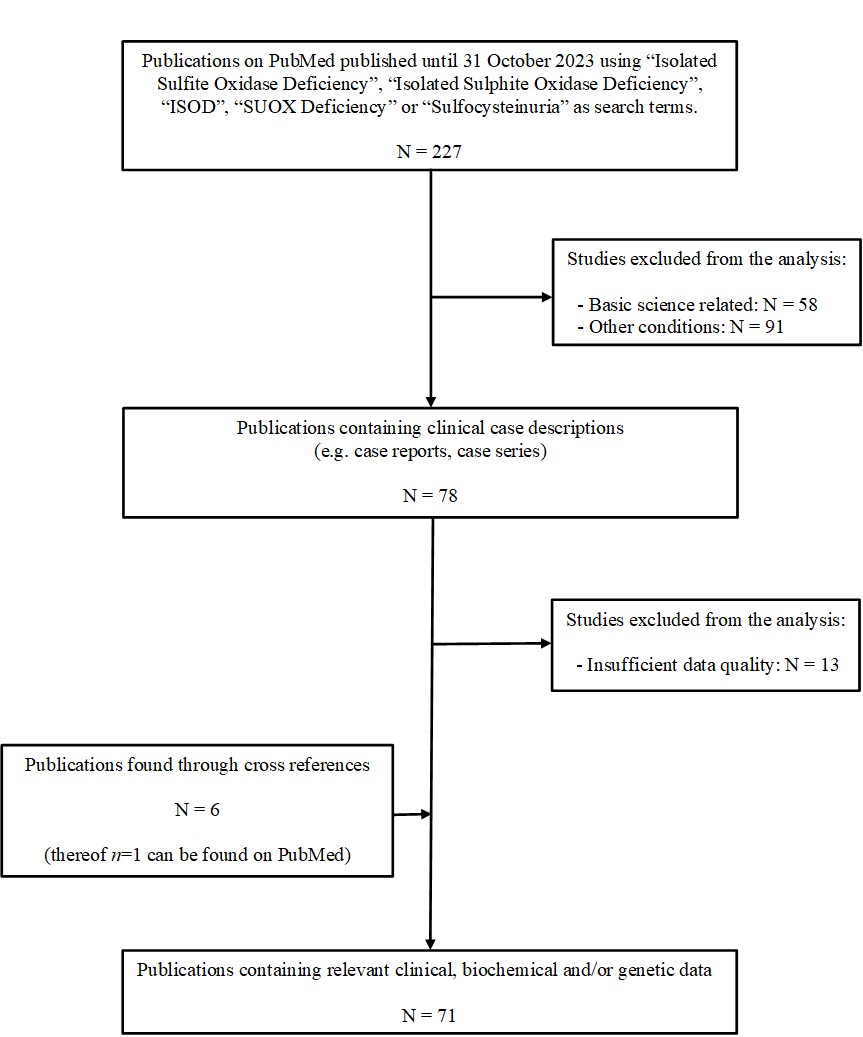

Supplement: S1 Fig — The reports analyzed were published up to 31 October 2023. ISOD, isolated sulfite oxidase deficiency. (JPG) [file pone.0323043.s001.jpg]

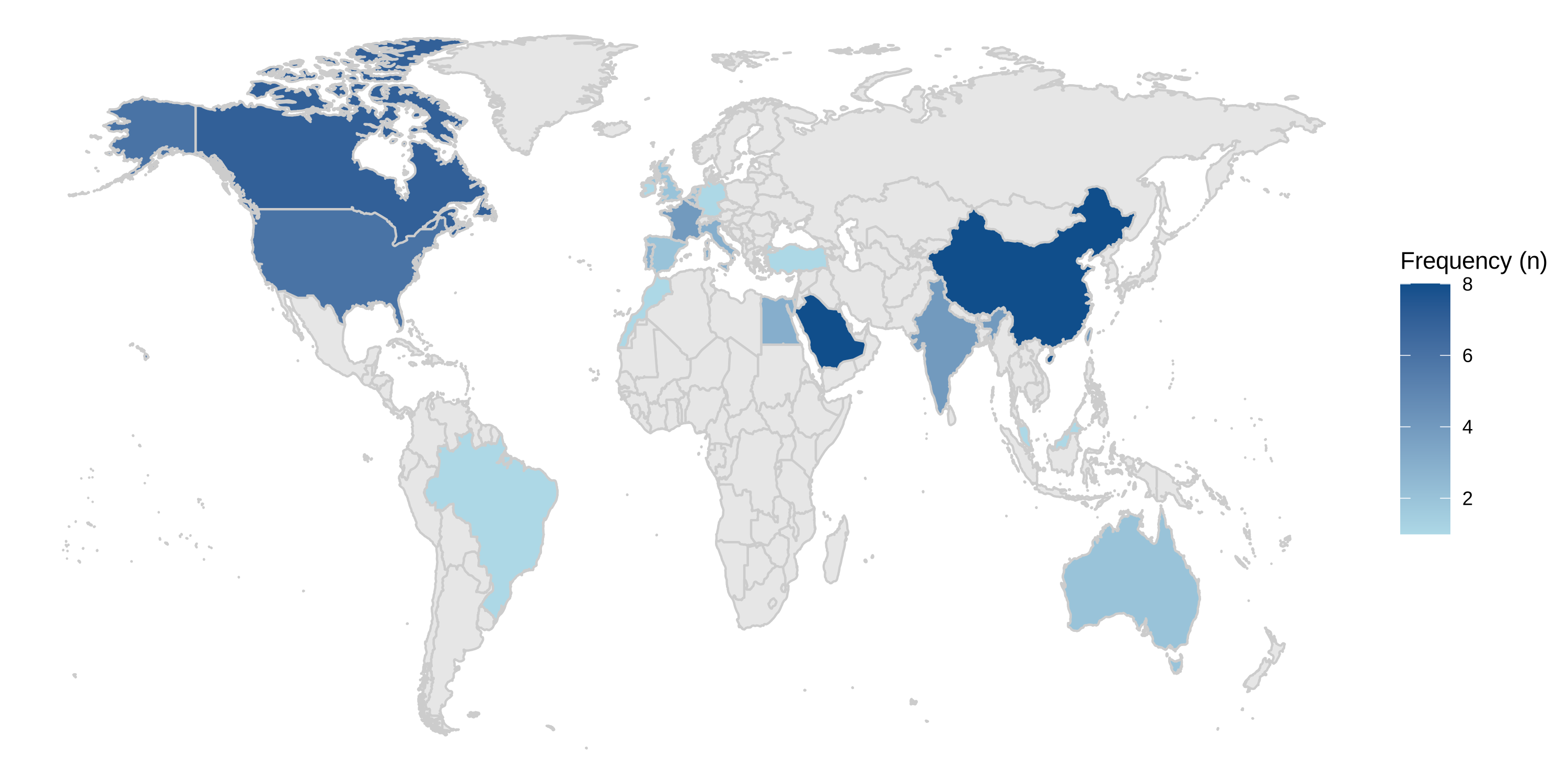

Supplement: S2 Fig — ISOD exhibited a panethnic distribution with hotspots in China, Saudi Arabia, Canada and the USA. Blue scales indicate the number of affected individuals per country. ISOD, isolated sulfite oxidase deficiency. (TIF) [file pone.0323043.s002.tif]

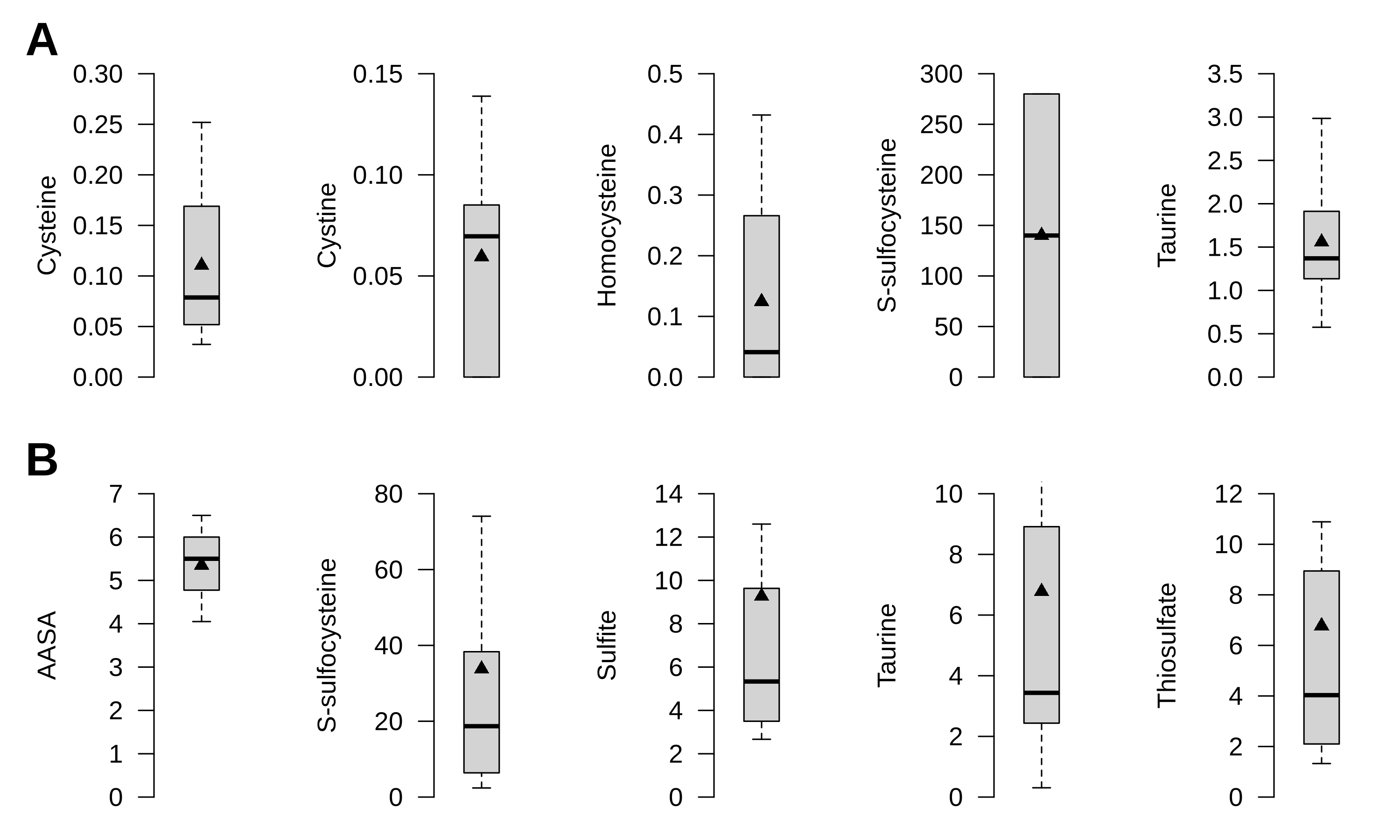

Supplement: S3 Fig — Numeric values of the ordinate refer to fold-change of mean of the respective reference range as relative values for each parameter. (A) Biochemical parameters as determined in plasma. (B) Biochemial parameters as determined in urine. For biochemical parameters the following outliers were not visualized due to reasons of graphical clarity: S-sulfocytsteine: 170; sulfite: 31; thiosulfate: 260; taurine: 20. Data are shown as median (black thick line) and mean (triangle), length of the box corresponds to the interquartile range (IQR), upper and lower whiskers correspond to max. 1.5 x IQR. AASA, α-aminoadipic semialdehyde; IQR, interquartile range; ISOD, isolated sulfite oxidase deficiency. (TIF) [file pone.0323043.s003.tif]

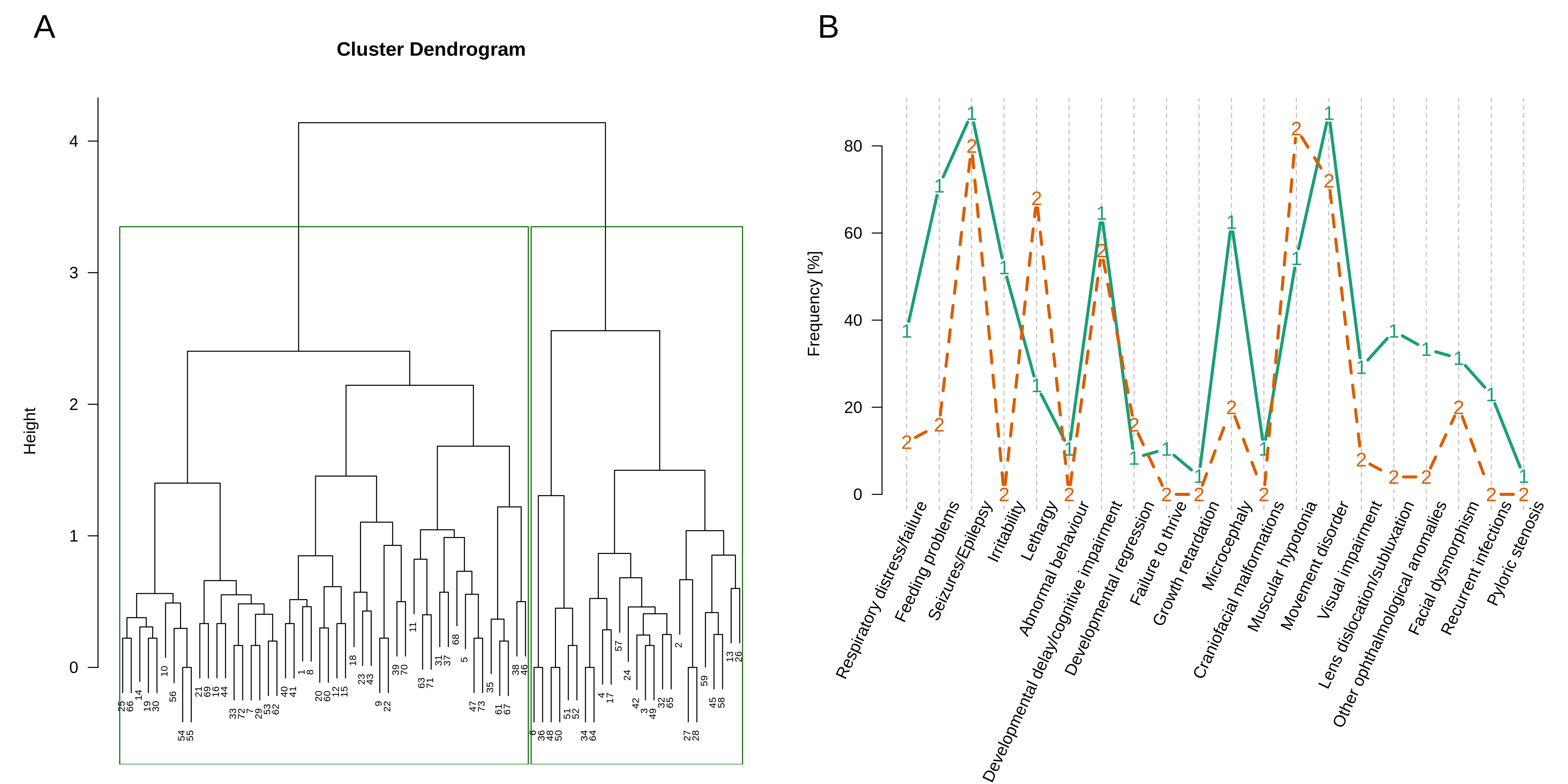

Supplement: S4 Fig — (A) Dendrogram: Clinical symptom clustering. The dendrogram enabled the grouping of clinical features into two groups. (B) Relativ frequency of symptoms per group. The two mathematically defined groups exhibited similar disease features in varying frequency, indicating a spectrum of increasing phenotypic severity in ISOD. ISOD, isolated sulfite oxidase deficiency. (TIF) [file pone.0323043.s004.tif]

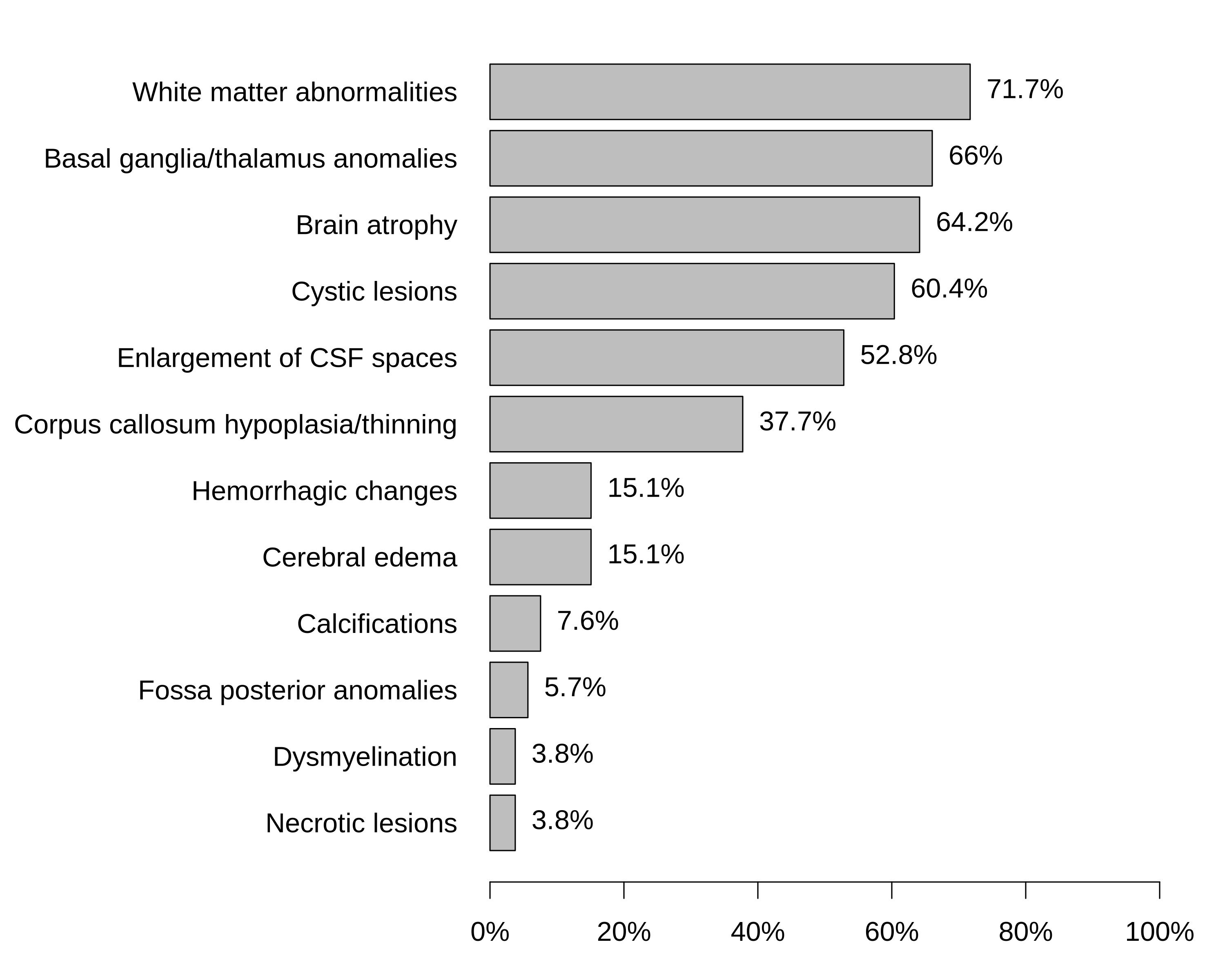

Supplement: S5 Fig — In individual probands two or more signs or symptoms may have been reported (N = 53). ISOD; isolated sulfite oxidase deficiency. (TIF) [file pone.0323043.s005.tif]
