# Supplementary material for: Ultra-orphan diseases: A cross-sectional quantitative analysis of the natural history of isolated sulfite oxidase deficiency
Source: PLoS One. 2025 May 29;20(5):e0323043. doi: 10.1371/journal.pone.0323043 (PMC12122042; doi:10.1371/journal.pone.0323043)
Supplement: S1 Table — (DOCX) [file pone.0323043.s006.docx]

**S1 Table**

**Inventory of publications on individuals with ISOD included in the present analysis.**

| **Publications containing relevant clinical, biochemical and/or genetic information*** | | **Included patients** | **Duplicate patients** |
| --- | --- | --- | --- |
| 1 | Li, J.-T. *et al.* (2022) ‘Mutation analysis of SUOX in isolated sulfite oxidase deficiency with ectopia lentis as the presenting feature: insights into genotype–phenotype correlation’, *Orphanet Journal of Rare Diseases*, 17, 392. <https://doi.org/10.1186/s13023-022-02544-x>. | 1 |  |
| 2 | Zhang, R. *et al.* (2022) ‘Whole exome sequencing identified a homozygous novel mutation in SUOX gene causes extremely rare autosomal recessive isolated sulfite oxidase deficiency’, *Clinica Chimica Acta*, 532, pp. 115–122. <https://doi.org/10.1016/j.cca.2022.06.005>. | 1 |  |
| 3 | Pavel, A.M. *et al.* (2021) ‘Case Report: Electroencephalography in a neonate with isolated sulfite oxidase deficiency – a case report and literature review’, *HRB Open Research*, 4, 122. <https://doi.org/10.12688/hrbopenres.13442.1>. | 1 |  |
| 4 | Ergene, M. *et al.* (2021) ‘Severe isolated sulfide oxidase deficiency with a novel mutation’, *The Turkish Journal of Pediatrics*, 63(4), pp. 716–720. <https://doi.org/10.24953/turkjped.2021.04.021>. | 1 |  |
| 5 | Ren, Z. *et al.* (2021) ‘Very early neuroimages of sulfite oxidase deficiency mimicing severe hypoxic ischemic encephalopathy in a neonate’, *Pediatrics and Neonatology*, 62(4), pp. 443–444. <https://doi.org/10.1016/j.pedneo.2021.03.005>. | 1 |  |
| 6 | Owen, M.J. *et al.* (2021) ‘Postmortem whole-genome sequencing on a dried blood spot identifies a novel homozygous SUOX variant causing isolated sulfite oxidase deficiency’, *Cold Spring Harbor Molecular Case Studies*, 7(3), a006091. <https://doi.org/10.1101/mcs.a006091>. | 1 |  |
| 7 | Saini, A.G. and Attri, S.V. (2021) ‘Cholestatic Jaundice in Sulphite Oxidase Deficiency - An Unusual Association: Correspondence’, *Indian Journal of Pediatrics*, 88(6), pp. 628–629. <https://doi.org/10.1007/s12098-020-03560-0>. | 1 | Patient is also reported in #ref 10 |
| 8 | Zhao, J. *et al.* (2021) ‘Novel Compound Heterozygous Pathogenic Variants in SUOX Cause Isolated Sulfite Oxidase Deficiency in a Chinese Han Family’, *Frontiers in Genetics*, 12, 607085. <https://doi.org/10.3389/fgene.2021.607085>. | 1 |  |
| 9 | Du, P. *et al.* (2021) ‘Identification of a novel SUOX pathogenic variants as the cause of isolated sulfite oxidase deficiency in a Chinese pedigree’, *Molecular Genetics & Genomic Medicine*, 9(2), e1590. <https://doi.org/10.1002/mgg3.1590>. | 1 |  |
| 10 | Ong, A.S.K. *et al.* (2021) ‘Cholestatic Jaundice in Sulphite Oxidase Deficiency - An Unusual Association’, *Indian Journal of Pediatrics*, 88(1), pp. 64–66. <https://doi.org/10.1007/s12098-020-03428-3>. | 1 | Patient is also reported in #ref 7 |
| 11 | Mhanni, A.A. *et al.* (2020) ‘Isolated sulfite oxidase deficiency: a founder mutation’, *Cold Spring Harbor Molecular Case Studies*, 6(6), a005900. <https://doi.org/10.1101/mcs.a005900>. | 2 |  |
| 12 | Scramstad, C., Moffatt, H. and Rafay, M.F. (2020) ‘Teaching NeuroImages: Early imaging of sulfite oxidase deficiency mimics severe hypoxic ischemic encephalopathy’, *Neurology*, 95(13), pp. e1913–e1914. <https://doi.org/10.1212/WNL.0000000000010258>. | 1 |  |
| 13 | Sharawat, I.K. *et al.* (2020) ‘Metabolic crisis after trivial head trauma in late-onset isolated sulfite oxidase deficiency: Report of two new cases and review of published patients’, *Brain & Development*, 42(2), pp. 157–164. <https://doi.org/10.1016/j.braindev.2019.11.003>. | 2 |  |
| 14 | Tian, M. *et al.* (2019) ‘Stable clinical course in three siblings with late-onset isolated sulfite oxidase deficiency: a case series and literature review’, *BMC Pediatrics*, 19, 510. <https://doi.org/10.1186/s12887-019-1889-5>. | 3 |  |
| 15 | Bender, D. *et al.* (2019) ‘Impaired mitochondrial maturation of sulfite oxidase in a patient with severe sulfite oxidase deficiency’, *Human Molecular Genetics*, 28(17), pp. 2885–2899. <https://doi.org/10.1093/hmg/ddz109>. | 1 |  |
| 16 | Boyer, M. *et al.* (2019) ‘Isolated Sulfite Oxidase Deficiency: Response to Dietary Treatment in a Patient with Severe Neonatal Presentation’, *Journal of Inborn Errors of Metabolism and Screening*, 7, e20190001. <https://doi.org/10.1590/2326-4594-jiems-2019-0001>. | 1 | Patient is also reported in #ref 23 |
| 17 | Salas, S. and Arca, G. (2018) ‘Sospecha diagnóstica por imagen de déficit de sulfito oxidase’, *Anales De Pediatria*, 88(4), pp. 230–231. <https://doi.org/10.1016/j.anpedi.2017.10.009>. | 1 |  |
| 18 | Claerhout, H. *et al.* (2018) ‘Isolated sulfite oxidase deficiency’, *Journal of Inherited Metabolic Disease*, 41(1), pp. 101–108. <https://doi.org/10.1007/s10545-017-0089-4>. | 3 | 1 patient is also reported in #ref 54 |
| 19 | Brumaru, D. *et al.* (2017) ‘A compound heterozygote case of isolated sulfite oxidase deficiency’, *Molecular Genetics and Metabolism Reports*, 12, pp. 99–102. <https://doi.org/10.1016/j.ymgmr.2017.06.009>. | 1 |  |
| 20 | Lee, H.-F. *et al.* (2017) ‘Prenatal brain disruption in isolated sulfite oxidase deficiency’, *Orphanet Journal of Rare Diseases*, 12, 115. <https://doi.org/10.1186/s13023-017-0668-3>. | 1 |  |
| 21 | Zaki, M.S. *et al.* (2016) ‘Molybdenum cofactor and isolated sulphite oxidase deficiencies: Clinical and molecular spectrum among Egyptian patients’, *European journal of paediatric neurology,* 20(5), pp. 714–722. <https://doi.org/10.1016/j.ejpn.2016.05.011>. | 3 |  |
| 22 | Relinque, B. *et al.* (2015) ‘Isolated sulfite oxidase deficiency’, *Journal of Neonatal-Perinatal Medicine,* 8(1), pp. 53-55. <https://doi.org/10.3233/NPM-15814029>. | 1 |  |
| 23 | Boyer, M. *et al.* (2015) ‘Program and Abstracts for the SIMD Annual Meeting: Isolated sulfite oxidase deficiency: Neonatal presentation with additional biochemical findings and diet therapy’, *Molecular Genetics and Metabolism*, 114, pp. 295–379. <https://doi.org/10.1016/j.ymgme.2014.12.308>. | 1 | Patient is also reported in #ref 16 |
| 24 | Chen, L.-W., Tsai, Y.-S. and Huang, C.-C. (2014) ‘Prenatal multicystic encephalopathy in isolated sulfite oxidase deficiency with a novel mutaion’, *Pediatric Neurology*, 51(1), pp. 181–182. <https://doi.org/10.1016/j.pediatrneurol.2014.03.010>. | 1 |  |
| 25 | Holder, J.L. *et al.* (2014) ‘Infantile spasms and hyperekplexia associated with isolated sulfite oxidase deficiency’, *JAMA Neurology*, 71(6), pp. 782–784. <https://doi.org/10.1001/jamaneurol.2013.5083>. | 1 |  |
| 26 | Westerlinck, H. *et al.* (2014) ‘Sulfite oxidase deficiency in a newborn’, *Journal of the Belgian Society of Radiology,* 97(2), pp. 113–114. <https://doi.org/10.5334/jbr-btr.40>. | 1 |  |
| 27 | Rocha, S. *et al.* (2014) ‘Sulfite oxidase deficiency-an unusual late and mild presentation’, *Brain & Development*, 36(2), pp. 176–179. <https://doi.org/10.1016/j.braindev.2013.01.013>. | 1 |  |
| 28 | Bosley, T.M. *et al.* (2014) ‘Neurologic injury in isolated sulfite oxidase deficiency’, *The Canadian Journal of Neurological Sciences,* 41(1), pp. 42–48. <https://doi.org/10.1017/s0317167100016243>. | 6 | 5 patients are also reported elsewhere:  - 1 patient is also reported in #ref 43, #ref 44  - 2 patients are also reported in #ref 31, #ref 44  - 1 patient is also reported in #ref 31  - 1 patient is also reported in #ref 44 |
| 29 | Cho, S.Y. *et al.* (2013) ‘Microarray analysis unmasked paternal uniparental disomy of chromosome 12 in a patient with isolated sulfite oxidase deficiency’, *Clinica Chimica Acta*, 426, pp. 13–17. <https://doi.org/10.1016/j.cca.2013.08.013>. | 1 |  |
| 30 | Del Rizzo, M. *et al.* (2013) ‘Metabolic stroke in a late-onset form of isolated sulfite oxidase deficiency’, *Molecular Genetics and Metabolism*, 108(4), pp. 263–266. <https://doi.org/10.1016/j.ymgme.2013.01.011>. | 1 |  |
| 31 | Salih, M.A. *et al.* (2013) ‘Preimplantation genetic diagnosis in isolated sulfite oxidase deficiency’, *The Canadian Journal of Neurological Sciences*, 40(1), pp. 109–112. <https://doi.org/10.1017/s0317167100013081>. | 3 | 3 patients are also reported elsewhere:  - 2 patients are also reported in #ref 28, #ref 44  - 1 patient is also reported in #ref 28 |
| 32 | Mills, P.B. *et al.* (2012) ‘Urinary AASA excretion is elevated in patients with molybdenum cofactor deficiency and isolated sulphite oxidase deficiency’, *Journal of Inherited Metabolic Disease*, 35(6), pp. 1031–1036. <https://doi.org/10.1007/s10545-012-9466-1>. | 2 | 1 patient is also reported in #ref 39 |
| 33 | Huang, Y.-L. *et al.* (2012) ‘^99^mTc-ethyl cysteinate dimer cranial single-photon emission computed tomography and serial cranial magnetic resonance imaging in a girl with isolated sulfite oxidase deficiency’, *Pediatric Neurology*, 47(1), pp. 44–46. <https://doi.org/10.1016/j.pediatrneurol.2012.03.012>. | 1 |  |
| 34 | Balasubramaniam, S. *et al.* (2012) ‘Isolated sulﬁte oxidase deﬁciency, a rare neurodegenerative disorder which mimics hypoxic-ischemic encephalopathy’, *Journal of Pediatric Neurology,* 10(1), pp.67-71. https://doi.org/10.3233/jpn-2011-0509. | 1 |  |
| 35 | Bindu, P.S. *et al.* (2011) ‘Clinical and imaging observations in isolated sulfite oxidase deficiency’, *Journal of Child Neurology*, 26(8), pp. 1036–1040. <https://doi.org/10.1177/0883073811401399>. | 2 |  |
| 36 | Palumbo, E. *et al.* (2010) ‘PP-45. Isolated sulfite oxidase deficiency: A case report’, *Early Human Development*, 86, p. S36. <https://doi.org/10.1016/j.earlhumdev.2010.09.098>. | 1 |  |
| 37 | Sass, J.O. *et al.* (2010) ‘Functional deficiencies of sulfite oxidase: Differential diagnoses in neonates presenting with intractable seizures and cystic encephalomalacia’, *Brain & Development*, 32(7), pp. 544–549. <https://doi.org/10.1016/j.braindev.2009.09.005>. | 1 |  |
| 38 | Hoffmann, C. *et al.* (2007) ‘Magnetic resonance imaging and magnetic resonance spectroscopy in isolated sulfite oxidase deficiency’, *Journal of Child Neurology*, 22(10), pp. 1214–1221. <https://doi.org/10.1177/0883073807306260>. | 2 |  |
| 39 | Basheer, S.N. *et al.* (2007) ‘Isolated sulfite oxidase deficiency in the newborn: lactic acidaemia and leukoencephalopathy’, *Neuropediatrics*, 38(1), pp. 38–41. <https://doi.org/10.1055/s-2007-981484>. | 1 | Patient is also reported in #ref 32 |
| 40 | Eichler, F. *et al.* (2006) ‘Proton magnetic resonance spectroscopy and diffusion-weighted imaging in isolated sulfite oxidase deficiency’, *Journal of Child Neurology*, 21(9), pp. 801–805. <https://doi.org/10.1177/08830738060210090601>. | 1 | Patient is also reported in #ref 42 |
| 41 | Hobson, E.E. *et al.* (2005) ‘Isolated sulphite oxidase deficiency mimics the features of hypoxic ischaemic encephalopathy’, *European Journal of Pediatrics*, 164(11), pp. 655–659. <https://doi.org/10.1007/s00431-005-1729-5>. | 1 |  |
| 42 | Tan, W.-H. *et al.* (2005) ‘Isolated Sulfite Oxidase Deficiency: A Case Report With a Novel Mutation and Review of the Literature’, *Pediatrics*, 116(3), pp. 757–766. <https://doi.org/10.1542/peds.2004-1897>. | 1 | Patient is also reported in #ref 40 |
| 43 | Seidahmed, M.Z. *et al.* (2005) ‘Total truncation of the molybdopterin/dimerization domains of SUOX protein in an Arab family with isolated sulfite oxidase deficiency’, *American Journal of Medical Genetics,* 136A(2), pp. 205–209. <https://doi.org/10.1002/ajmg.a.30796>. | 1 | Patient is also reported in #ref 28 and #ref 44 |
| 44 | Rashed, M.S. *et al.* (2005) ‘Determination of urinary S-sulphocysteine, xanthine and hypoxanthine by liquid chromatography-electrospray tandem mass spectrometry’, *Biomedical Chromatography*, 19(3), pp. 223-230. <https://doi.org/10.1002/bmc.439>. | 6 | 6 patients are also reported elsewhere:  - 1 patient is also reported in #ref 28  - 2 patients are also reported in. #ref 28, #ref 31  - 1 patient is also reported in #ref 28, #ref 43  - 2 patients are also reported in #ref 45 |
| 45 | Eyaid, W.M. *et al.* (2005) ‘An inborn error of metabolism presenting as hypoxic-ischemic insult’, *Pediatric Neurology*, 32(2), pp. 134–136. <https://doi.org/10.1016/j.pediatrneurol.2004.07.010>. | 2 | 2 patients also reported in #ref 44 |
| 46 | Sass, J.O. *et al.* (2004) ‘New approaches towards laboratory diagnosis of isolated sulphite oxidase deficiency’, *Annals of Clinical Biochemistry*, 41(2), pp. 157–159. <https://doi.org/10.1258/000456304322880078>. | 2 | 2 patients are also reported elsewhere:  - 1 patient also reported in #ref 49; - 1 patient also reported in #ref 52 |
| 47 | Schiaffino, M.C. *et al.* (2004) ‘Isolated sulphite oxidase deficiency: clinical and biochemical features in an Italian patient’, *Journal of Inherited Metabolic Disease*, 27(1), pp. 101–102. <https://doi.org/10.1023/b:boli.0000016674.61073.e4>. | 1 |  |
| 48 | Chan, K.Y. *et al.* (2002) ‘Infantile isolated sulphite oxidase deficiency in a Chinese family: a rare neurodegenerative disorder’, *Hong Kong Medical Journal*, 8(4), pp. 279–282. | 1 |  |
| 49 | Lee, H.F. *et al.* (2002) ‘A novel mutation in neonatal isolated sulphite oxidase deficiency’, *Neuropediatrics*, 33(4), pp. 174–179. <https://doi.org/10.1055/s-2002-34491>. | 1 | Patient is also reported in #ref 46 |
| 50 | Johnson, J.L. *et al.* (2002) ‘Isolated sulfite oxidase deficiency: identification of 12 novel SUOX mutations in 10 patients’, *Human Mutation*, 20(1), 74. <https://doi.org/10.1002/humu.9038>. | 1 | Patient is also reported in #ref 58 and #ref 61 |
| 51 | Johnson, J.L. *et al.* (2002) ‘Isolated sulfite oxidase deficiency: mutation analysis and DNA-based prenatal diagnosis’, *Prenatal Diagnosis*, 22(5), pp. 433–436. <https://doi.org/10.1002/pd.335>. | 1 |  |
| 52 | Dublin, A.B., Hald, J.K. and Wootton-Gorges, S.L. (2002) ‘Isolated sulfite oxidase deficiency: MR imaging features’, *American journal of neuroradiology*, 23(3), pp. 484–485. | 1 | Patient is also reported in #ref 46 |
| 53 | Lam, C.-W. *et al.* (2002) ‘DNA-based diagnosis of isolated sulfite oxidase deficiency by denaturing high-performance liquid chromatography’, *Molecular Genetics and Metabolism*, 75(1), pp. 91–95. <https://doi.org/10.1006/mgme.2001.3267>. | 1 |  |
| 54 | Touati, G. *et al.* (2000) ‘Dietary therapy in two patients with a mild form of sulphite oxidase deficiency. Evidence for clinical and biological improvement’, *Journal of Inherited Metabolic Disease*, 23(1), pp. 45–53. <https://doi.org/10.1023/a:1005646813492>. | 1 | Patient is also reported in #ref 18 |
| 55 | Edwards, M.C. *et al.* (1999) ‘Isolated sulﬁte oxidase deﬁciency: review of two cases in one family’, *Ophthalmology,* 106(10), pp. 1957-1961. <https://doi.org/10.1016/S0161-6420(99)90408-6>. | 1 |  |
| 56 | Garrett, R.M. *et al.* (1998) ‘Human sulfite oxidase R160Q: Identification of the mutation in a sulfite oxidase-deficient patient and expression and characterization of the mutant enzyme’, *Proceedings of the National Academy of Sciences of the United States of America*, 95(11), pp. 6394–6398. <https://doi.org/10.1073/pnas.95.11.6394>. | 1 | Patient is also reported in #ref 59 and #ref 61 |
| 57 | Goh, A. and Lim, K. (1997) ‘Sulphite oxidase deficiency-a report of two siblings’, *Singapore medical journal*, 38(9), pp. 391-394. | 1 |  |
| 58 | Rupar, C.A. *et al.* (1996) ‘Isolated sulfite oxidase deficiency’, *Neuropediatrics*, 27(6), pp. 299–304. <https://doi.org/10.1055/s-2007-973798>. | 1 | Patient is also reported in #ref 50 and #ref 61 |
| 59 | Cole, D.E.C. and Evrovski, J. (1996) ‘Screening for sulfite oxidase deficiency with urinary thiosulfate/sulfate ratios determined by anion chromatography’, *Clinical Chemistry*, 42(4), pp. 654–655. | 1 | Patient is also reported in #ref 56 and #ref 61 |
| 60 | Barbot, C. *et al.* (1995) ‘A mild form of infantile isolated sulphite oxidase deficiency’, *Neuropediatrics*, 26(6), pp. 322–324. <https://doi.org/10.1055/s-2007-979783>. | 1 |  |
| 61 | Johnson, J.L. and Rajagopalan, R.V. (1995) ‘An HPLC assay for detection of elevated urinary S-sulphocysteine, a metabolic marker of sulphite oxidase deficiency’, *Journal of Inherited Metabolic Disease*, 18(1), pp. 40-47. <https://doi.org/10.1007/BF00711371>. | 2 | 2 patients are also reported elsewhere:  - 1 patient is also reported in #ref 50 and #ref 58  - 1 patient is also reported in #ref 56 and #ref 59 |
| 62 | Amiel, J. *et al.* (1994) ‘Déficit en sulfite oxydase se présentant comme un syndrome de Leigh’, *Archives De Pediatrie: Organe Officiel De La Societe Francaise De Pediatrie*, 1(11), pp. 1023–1027. | 1 |  |
| 63 | Vilarinho, L. *et al.* (1994) ‘Citrullinaemia and isolated sulphite oxidase deficiency in two siblings’, *Journal of Inherited Metabolic Disease*, 17(5), pp. 638–639. <https://doi.org/10.1007/BF00711610>. | 1 |  |
| 64 | van der Klei-van Moorsel, J.M. *et al.* (1991) ‘Infantile isolated sulphite oxidase deficiency: Report of a case with negative sulphite test and normal sulphate excretion’, *European Journal of Pediatrics*, 150(3), pp. 196–197. <https://doi.org/10.1007/BF01963565>. | 1 |  |
| 65 | Tardy, P. *et al.* (1989) ‘Attempt at therapy in sulphite oxidase deficiency’, *Journal of Inherited Metabolic Disease*, 12(1), pp. 94–95. <https://doi.org/10.1007/BF01805537>. | 1 |  |
| 66 | Brown, G.K. *et al.* (1989) ‘Sulfite oxidase deficiency: clinical, neuroradiologic, and biochemical features in two new patients’, *Neurology*, 39(2), pp. 252–257. <https://doi.org/10.1212/wnl.39.2.252>. | 2 |  |
| 67 | Vianey-Liaud, C. *et al.* (1988) ‘A new case of isolated sulphite oxidase deficiency with rapid fatal outcome’, *Journal of inherited metabolic disease*, 11(4), pp. 425-426. <https://doi.org/10.1007/BF01800433>. | 1 |  |
| 68 | Johnson, J.L. and Rajagopalan, K.V. (1976) ‘Human sulfite oxidase deficiency. Characterization of the molecular defect in a multicomponent system’, *The Journal of Clinical Investigation*, 58(3), pp. 551–556. <https://doi.org/10.1172/JCI108500>. | 1 | Patient is also reported in #ref 69, #ref 70, #ref 71 |
| 69 | Rosenblum, W.I. (1968) ‘Neuropathologic changes in a case of sulfite oxidase deficiency’, *Neurology*, 18(12), pp. 1187–1196. <https://doi.org/10.1212/wnl.18.12.1187>. | 1 | Patient is also reported in #ref 68, #ref 70, #ref 71 |
| 70 | Irreverre, F. *et al.* (1967) ‘Sulfite oxidase deficiency: Studies of a patient with mental retardation, dislocated ocular lenses, and abnormal urinary excretion of S-sulfo-l-cysteine, sulfite, and thiosulfate’, *Biochemical Medicine*, 1(2), pp. 187–217. <https://doi.org/10.1016/0006-2944(67)90007-5>. | 1 | Patient is also reported in #ref 68, #ref 69, #ref 71 |
| 71 | Mudd, S.H., Irreverre, F. and Laster, L. (1967) ‘Sulfite oxidase deficiency in man: Demonstration of the enzymatic defect’, *Science*, 156(3782), pp. 1599–1602. <https://doi.org/10.1126/science.156.3782.1599>. | 1 | Patient is also reported in #ref 68, #ref 69, #ref 70 |
|  | **Number of patients included in this analysis** | **74** | |

*Articles are listed in descending order of their respective year of publication. ISOD, isolated sulfite oxidase deficiency.
