# Supplementary material for: Ultra-orphan diseases: A cross-sectional quantitative analysis of the natural history of isolated sulfite oxidase deficiency
Source: PLoS One. 2025 May 29;20(5):e0323043. doi: 10.1371/journal.pone.0323043 (PMC12122042; doi:10.1371/journal.pone.0323043)
Supplement: S2 Table — (DOCX) [file pone.0323043.s007.docx]

**S2 Table**

**Biochemical and survival characteristics of the ISOD study population.**

| **Variable** | **N** | **Mean +/- SD** | **Median** | **IQR** | **Min** | **Max** | **Correlation analyses** | |
| --- | --- | --- | --- | --- | --- | --- | --- | --- |
| Biochemical variable [plasma]  (relative value of mean of normal control) |  |  | | | | | Age at onset  (p-value) | Survival  (p-value) |
| Cysteine | 4 | 0.11 +/- 0.10 | 0.08 | 0.06 - 0.13 | 0.03 | 0.25 | 0.69 | 0.14 |
| Cystine | 13 | 0.06 +/- 0.05 | 0.07 | 0.00 - 0.09 | 0.00 | 0.14 | 0.33 | 0.69 |
| Homocysteine | 22 | 0.12 +/- 0.15 | 0.04 | 0.00 - 0.25 | 0.00 | 0.43 | 0.02 | 0.25 |
| S-sulfocysteine | 2 | 140.00 +/- 197.99 | 140.00 | 70.00 - 210.00 | 0.00 | 280.00 | n/a | 1.00 |
| Taurine | 6 | 1.56 +/- 0.82 | 1.37 | 1.17 - 1.80 | 0.58 | 2.98 | 0.59 | 0.58 |
| Homocysteine (µmol/L) | 22 | 1.05 +/- 1.33 | 0.30 | 0.00 – 2.08 | 0.00 | 3.74 | 0.02 | 0.25 |
| Biochemical variable [urine]  (relative value of mean of normal control) |  |  | | | | | | |
| AASA | 3 | 5.35 +/- 1.23 | 5.50 | 4.78 - 6.00 | 4.05 | 6.50 | n/a | 0.14 |
| Sulfite | 7 | 9.27 +/- 9.99 | 5.33 | 3.50 - 9.63 | 2.67 | 30.60 | 0.28 | 0.31 |
| S-sulfocysteine | 27 | 29.23 +/- 36.67 | 16.62 | 6.02 - 34.10 | 2.40 | 170.00 | 0.32 | 0.24 |
| Taurine | 9 | 6.77 +/- 6.20 | 3.44 | 2.44 - 8.91 | 0.31 | 19.83 | 0.39 | 0.28 |
| Thiosulfate | 8 | 36.40 +/- 90.28 | 4.03 | 2.28 - 7.97 | 1.33 | 259.69 | 0.55 | 0.09 |
| Sulfite oxidase activity  (relative value of mean of normal controls) |  |  | | | | | | |
| fibroblasts | 19 | 0.00 +/- 0.00 | 0.00 | 0.00 - 0.00 | 0.00 | 0.00 | n/a | 1.00 |
| hepatocytes | 5 | 0.00 +/- 0.00 | 0.00 | 0.00 - 0.00 | 0.00 | 0.00 | n/a | 1.00 |
| Last reported age (months) | 72 | 31.61 +/- 37.97 | 16.00 | 3.00 – 48.50 | 0.01 | 204.0 | | |

AASA, α-aminoadipic semialdehyde; IQR, interquartile range; ISOD, isolated sulfite oxidase deficiency; max, maximum; min; minimum; n/a, not available/applicable; SD, standard deviation.
