# Supplementary material for: Ultra-orphan diseases: A cross-sectional quantitative analysis of the natural history of isolated sulfite oxidase deficiency
Source: PLoS One. 2025 May 29;20(5):e0323043. doi: 10.1371/journal.pone.0323043 (PMC12122042; doi:10.1371/journal.pone.0323043)
Supplement: S3 Table — (DOCX) [file pone.0323043.s008.docx]

**S3 Table**

**Reported genotypes of individuals with ISOD (N=39).**

| **Allele1 (cDNA)** | **Allele 1 (protein)** | **Allele 2 (cDNA)** | **Allele 2 (protein)** | **Last reported age (months)** | **Alive** |
| --- | --- | --- | --- | --- | --- |
| c.1302del |  | c.601A>C | p.I201L | 32 | no |
| c.400_403del |  | c.400_403del |  | 2.3 | no |
| c.520del |  | c.520del |  | 60 | no |
| c.520del |  | c.520del |  | 2.2 | yes |
| c.1313_1316del |  | c.1313_1316del |  | 13 | yes |
| c.1029C>G | p.Y343X | c.1235_1250del |  | 0.5 | no |
| c.1136A>G | p.K379R | c.1136A>G | p.K379R | 24 | yes |
| c.1200C>G | p.Y400X | c.1200C>G | p.Y400X | 10 | yes |
| c.1200C>G | p.Y400X | c.1200C>G | p.Y400X | 28 | yes |
| c.427C>A | p.H143N | c.427C>A | p.H143N | 30 | yes |
| c.1234_1235del |  | c.1234_1235del |  | 14 | no |
| c.1234_1235del |  | c.1234_1235del |  | 108 | yes |
| c.182T>C | p.L61P | c.182T>C | p.L61P | 60 | yes |
|  | p.E282X |  | p.D512Y | 1.9 | no |
|  | p.W393R |  | p.W393R | 204 | yes |
| c.590_595del |  | c.590_595del |  | 72 | yes |
| c.1096C>T | p.R366C | c.1376G>A | p.R459Q | 114 | yes |
| c.1096C>T | p.R366C | c.1376G>A | p.R459Q | 66 | yes |
| c.1096C>T | p.R366C | c.1376G>A | p.R459Q | 54 | yes |
| c.1382A>T | p.D461V | c.1382A>T | p.D461V | 16 | yes |
| c.1382A>T | p.D461V | c.1382A>T | p.D461V | 30 | yes |
| c.1201A>G | p.K401E | c.475G>T | p.E159X | 9 | no |
| c.713G>A | p.G2138Q | c.713G>A | p.G238Q | n/a | no |
| c.884G>A | p.G295E | c.884G>A | p.G295E | n/a | no |
| c.884G>A | p.G295E | c.884G>A | p.G295E | 30 | yes |
| c.1347_1350del |  | c.1521_1524del |  | 108 | no |
| c.1347_1350del |  | c.1521_1524del |  | 15 | no |
| c.1200C>G | p.Y400X | c.1574_1575ins26 |  | 6 | yes |
|  | p.R160Q |  | p.Y343X | 108 | yes |
| c.1390_1391del |  | c.1390_1391del |  | 0.5 | no |
| c.1084G>A | p.G362S | c.1084G>A | p.G362S | 54 | yes |
| c.1227G>A | p.W409X | c.1227G>A | p.W409X | 0.3 | no |
| c.1585C>T | p.R529X | c.1585C>T | p.R529X | 24 | no |
| c.1585C>T | p.R529X | c.1585C>T | p.R529X | 2 | no |
| c.1200C>G | p.Y400X | c.1549_1574dup |  | 18 | yes |
| c.1084G>A | p.G362S | c.302G>A | p.W101X | 6 | yes |
| c.1200C>G | p.Y400X | c.205G>C | p.A69P | 50 | no |
| c.1029C>G | p.Y343X | c.1029C>G | p.Y343X | 7 | yes |
| c.192dup |  | c.192dup |  | 4 | no |

ISOD, isolated sulfite oxidase deficiency; n/a, not available
